# Evaluating the Accuracy of Two Microleakage Assessment Methods for Mineral Trioxide Aggregate and Calcium-enriched Mixture Cement

**DOI:** 10.22037/iej.v12i4.17796

**Published:** 2017

**Authors:** Fatemeh Ayatollahi, Milad Hazeri Baqdad Abad, Seyed Hossein Razavi, Mahdi Tabrizizadeh, Reza Ayatollahi, Fatemeh Zarebidoki

**Affiliations:** a *Department of Endodontics, Dental School, Shahid Sadoughi University of Medical Sciences, Yazd ,Iran; *; b *Department of Orthodontics Dentistry, Dental School, Shahid Sadoughi University of Medical Sciences, Yazd, Iran; *; c *Department of oral and maxillofacial radiology, Dental Faculty, Yazd Shahid Sadoughi university of medical sciences. Yazd ,Iran;*; d * Dental School, Shahid Sadoughi University of Medical Sciences, Yazd, Iran;*; e *Department of Pediatric Dentistry, Dental School, Shahid Sadoughi University of Medical Sciences, Yazd, Iran*

**Keywords:** Calcium-enriched Mixture Cement, Marginal Adaptation, Microleakage, Mineral Trioxide Aggregate

## Abstract

**Introduction:**

Multiple methods for evaluating microleakage have been introduced over the years, but there has been no agreement as to which technique will give more accurate results. The aim of this study was to compare the accuracy and results of fluid filtration and marginal adaptation methods for mineral trioxide aggregate (MTA) and calcium-enriched mixture (CEM) cement apical plugs.

**Methods and Materials:**

A total of 250 single-rooted human teeth were collected. The teeth were decoronated, the root canals were prepared and open apex condition was stimulated by passing #1 to 4 Peeso Reamer drills from apical foramen. Five teeth were selected as the positive and negative controls and the rest of the samples were randomly allocated to two groups of MTA and CEM cement plugs. In each group, apical plug was placed into the canal. After the apical plugs were completely set, microleakage and marginal adaptation of the samples were evaluated using fluid filtration method and scanning electron microscopy (SEM), respectively. The obtained results were analyzed by independent-samples *t *test.

**Results:**

Gap between plug and dentin walls and air bubbles displacement was higher in MTA group compared to the CEM cement group, though this difference between MTA group and CEM cement group was not statistically significant. **Conclusion: **According to the obtained results, it seems that there is a direct relationship between the two methods of microleakage assessment.

## Introduction

Endodontic treatment of immature teeth with open apices is considered as a challenge in endodontics. As the apical region is wide and due to the absence of apical constriction, the determination of working length and condensation of gutta-percha in such teeth is very difficult [1-3]. Today, the use of apical plug is preferred over the traditional apexification method due to reduction in the number of treatment sessions and being less dependent on patient’s cooperation [4, 5]. 

Different materials including mineral trioxide aggregate (MTA), calcium-enriched mixture (CEM) cement, resorbable ceramics and dentin chips has been suggested for creating an artificial barrier and apexification in such teeth [1, 4, 6]. Due to favorable properties such as sealing ability, biocompatibility and high antibacterial properties MTA is commonly being used in this procedure [4].

CEM cement has clinical performance similar to MTA [7, 8]. Although the biocompatibility and sealing ability of CEM cement is similar to MTA, but it has some advantages over MTA including higher anti-bacterial properties and shorter setting time [9-11].

Clinical studies have shown that apical leakage is one of the main causes of failure of endodontic treatment [9].

On the other hand, several studies have highlighted the importance of dimensional stability as one of the essential characteristics for the root filling material [10]. The lack of dimensional stability, and subsequently the loss of marginal adaptation through creating a path for microorganisms’ penetration can cause microleakage and disruption of sealing ability of the material [10, 11].

Several studies conducted have shown that assessing marginal adaptation can be an indirect assessment of microleakage level [11-14], while others have reported that there is no relationship between marginal adaptation and sealing ability of a material [15, 16].

Considering the insufficient information in this area, this study aimed to investigate the correlation between marginal adaptation and sealing ability of MTA and CEM cement.

## Materials and Methods

This research was approved by research council of dentistry faculty of Yazd University of medical science. In order to perform this study, 250 single-rooted human teeth with single canals, closed apices and without severe caries, large coronal restoration or root curvature were chosen. For infection control and elimination of the remnants of periodontal soft tissues, the samples were placed in 5.25% sodium hypochlorite solution for 1 h and then were stored in normal saline until the experiment. In order to uniform the length of roots, dental crowns were cut by a diamond disc (010, Tizkavan, Tehran, Iran) with high-speed handpiece in such a way that ultimate length of roots was 13 mm. Working length was measured by a #15 K-file (Dentsply Maillefer, Ballaigues, Switzerland).

After the enlargement of coronal part of the canals by Gates-Glidden drills (Dentsply Maillefer, Ballaigues, Switzerland), the canals were cleaned by #40 K-file up and were flared up to #80.

During preparation, the canal was washed with 2.25% sodium hypochlorite solution. In the next step, in order to simulate the conditions of the open apex teeth, #1 to 4 Peeso Reamer drills (Dentsply, Maillefer, Tulsa, OK, USA) were passed orthogradely to create apical foramen with 1.3 mm diameter.

In compliance with standard protocol of canal debridement and to remove smear layer, all teeth were filled with 1 mL of 17% ethylene diamine tetraacetic acid (EDTA) (Ariadent, Tehran, Iran) for 3 min and then were rinsed by 5 mL of normal saline and dried by paper cones.

Five canals were filled with gutta-percha (Diadent, Chongju, Korea) without sealer were considered as the positive control. As negative controls, apex of 5 teeth was covered with sticky wax (Kerr, Berlin, Germany) and then two layers of nail varnish was applied on the surface of teeth.

Other prepared roots were randomly divided into two MTA and CEM cement groups containing 60 teeth in each group, apical plug was placed into the canal.

Group 1: powder and liquid of white MTA (Angelus, Londrina, PR, Brazil) were mixed according to the manufacturer's instructions, and the paste was inserted inside the canal orthogradely using MTA carrier (Dentsply Maillefer, Ballaigues, Switzerland) and was condensed using #3 and 4 hand pluggers (Dentsply Maillefer, Ballaigues, Switzerland) in such a way that the length of the entry was 5 mm shorter than the working length. Ultimately apical plug with a thickness of 5 mm remained after condensation. The thickness and density of the apical plug was examined with radiography and after placing a wet paper point into the canal, access cavity was filled by temporary restoration (Coltosol, Ariadent, Tehran, Iran). The samples were kept in incubator at 37^°^C with 100% humidity for 24 h. After 24 h, the temporary restorations were removed, the complete setting of MTA was checked and then the rest of canal space was filled with gutta-percha and AH-26 sealer (Dentsply DeTrey, Konstanz, Germany) using lateral condensation technique.

Group 2: CEM cement (Bionique Dent, Tehran, Iran) was mixed according to the manufacturer's instructions and similar to group 1, apical plug was placed into the canal. The samples were kept at 37^°^C and 100% humidity for 24 h.

***Fluid filtration technique***

**Figure 1:**
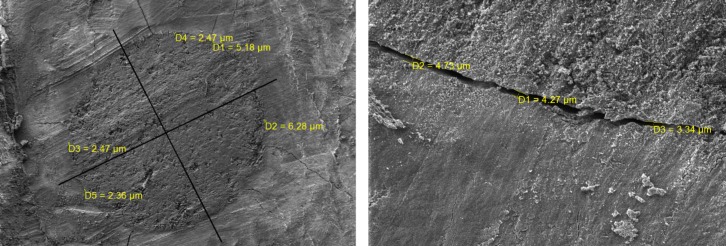
Scanning electron microscopic images of apical plug

In order to assess microleakage, prepared teeth were transferred to the device as described in the study by Moradi *et al.* [17]. Briefly, the device measures microleakage by standard fluid filtration method (Figure 1). To prepare samples for microleakage evaluation, samples except in the 2 mm of apical region were covered by 2 layers of nail varnish and Parafilm.

The apical part of the samples was attached to the latex tubing using Cyanoacrylate adhesive (Razi, Tehran, Iran). To connect the tube to the leakage evaluation system, the other end of latex tubing was connected to one of three-way plastic valves of adapter in the device. Other valve was connected to insulin syringes to create air bubbles and the third valve was connected to a standard micropipette. The other end of the micropipette connected to pressure transducer (by applying air pressure of 0.5 atmosphere to the Beaker containing tab water, the air pressure transforms to the water pressure). All routs (such as pipette, plastic tubes and syringes) were filled with distilled water.

After connecting the samples and creation of small air bubble inside the micropipette and exerting pressure to the track (route), the amount of leakage was reported based on the amount of bubble displacement within the micropipette and its conversion into the volume of displaced fluid in μL/min/cmH_2_O.

All measurements were performed three times and the average value was reported as the microleakage value. 

***Evaluation of marginal adaptation***

In order to evaluate marginal adaptation in each group, the roots were cut by diamond disc horizontally with plenty of water. The teeth were cut at 1 and 2 mm away from the apex in order to create a sample with a thickness of 1 mm. The samples were prepared for further assessment by scanning electron microscope (SEM) (TESCAN VEGA3, Czesh Republic) in Physics Laboratory at the University of Yazd.

The obtained image was divided into 4 equal parts and in each part the maximum amount of gap between apical plug and dentine was measured by relevant software. Finally, the average of these four values obtained from each sample was reported as the final gap width of each sample.

The collected data were analyzed by independent samples *t* test and using the statistical software SPSS 18 (SPSS version 18.0, SPSS, Chicago, IL, USA). *P*-value less than 0.05 was considered significant.

## Results

Table 1 shows the amount of air bubble displacement (movement) and the amount of gap between the two experimental groups. As seen in table 1, in the MTA apical plug group the amount of bubble displacement as well as the gap between plug and dentin wall of the canal is higher than CEM cement apical plug group. Although these differences were not statistically significant. All negative and positive control groups showed results as expected.

## Discussion

The aim of this study was to investigate the correlation between the results of microleakage and marginal adaptation. To perform this study, MTA and CEM cement was used to make apical plug. These materials are common used materials in this method and their beneficial properties such as sealing ability, biocompatibility, and antibacterial properties have been proven [7, 18-23]. Nowadays, synthetic apical plug method has become increasingly popular for the treatment of immature necrotic teeth [4, 5].

It has been shown that microleakage plays a key role in endodontic treatment failure [24]. In addition, the dimensional stability is one of the essential properties for root end filling materials [10]. Material shrinkage during setting causes loss of marginal adaptation and creates a gap between restorative material and the dentin walls and thus provides a path for the transmission of liquids and microorganisms. Therefore, study of marginal adaptation of materials can be an indirect evaluation of sealing ability of materials [10].

However, there are many disagreements on this issue. For instance, Stabholze *et al. *[11], Tewari* et al.* [14], Shani* et al.* [12] and Torabinezhad *et al.* [13] in separate studies have shown that there is a direct relationship between microleakage and marginal adaptation while this results was inconsistent with the results of the study by Xavier* et al. *[15] considering these disagreements and due to the lack of sufficient information.

In order to assess the microleakage, fluid filtration method was used in this study. In this method, the sealing ability of the material is measured by the movement of air bubble under pressure within a capillary tube [25]. This method has several advantages over other microleakage assessments methods. For example, this method is a quantitative method and unlike the conventional method of dye penetration, the samples are not destroyed and hence the sample can be studied over time. In this method, there is no molecular marker and hence the molecular marker-related errors can be avoided. Besides, recording even very small volume of microleakage increases the sensitivity of this method [26-28]. 

**Table 1:** The mean (SD) of microleakage (10^⁻^^4^×µL/min/cmH_2_O) and gap value (µm

**Material**	**Mean (SD) of microleakage (10** **⁻** **4** **×µ** **L/min/cmH**_2_ **O)**	**Mean (SD)of gap value (µm)**
**MTA**	34.051 (1.201)	5.019 (0.227)
**CEM cement**	32.778 (±1.201)	4.846 (0.231)
***P*** **-value**	0.455	0.593

A comparative study by Stabholze *et al. *[11] on four retrograde filling material and a comparative study by Tewari *et al. *[14] on retrograde and orthograde amalgam, confirmed the relationship between the marginal adaptation and the sealing ability using dye penetration method. 

However, Xavier *et al. *and Costa *et al. *[15, 29] in two studies evaluated the marginal adaptation and dye penetration of root end filling material and reported that there is no relationship between these two factors. This contradictory result can be due to the use of resin replica and indirect assessment of the amount of gap. Verissimo *et al. *[28] believes that disparity between the reported results may be due to the lack of standard assessment methods.

As previously mentioned, the results of this study demonstrate a direct correlation between the marginal adaptation and the sealing ability. The results of this study can be attributed to the properties of the used materials. MTA and CEM cement are hydrophilic materials that can absorb liquids while setting process and acquirethe ability to penetrate into dentinal tubules and gaps. These characteristics, along with a slight expansion during setting process and production of crystalline hydroxyapatite in the border of material and dentin walls can provide an excellent sealing and marginal adaptation [8, 30, 31].

However, considering the disagreements among reported results, it is necessary to take the limitations of this study into account. The study of marginal adaptation only shows the gap between the plug and the dentin wall while microleakage occurs both inside of the apical plug and in the boundary between the plug and dentine wall. As a result, it is difficult to determine with certainty the relationship between marginal adaptation and sealing ability of the material. Nevertheless, the presence of gap and the lack of marginal adaptation can affect the sealing ability.

## Conclusion

Considering the obtained results and the condition of this study it seems that there is a direct relationship between marginal adaptation and sealing ability of the materials. However, in order to confirm these results, further studies using other methods of microleakage assessment seem necessary.
